# Transcriptome and metabolite profiles reveal differential molecular responses of wild and cultivated amaranth species to water deficit and salt stress

**DOI:** 10.1007/s00425-026-04927-x

**Published:** 2026-01-29

**Authors:** Ana P. Barba de la Rosa, Jose Cetz, Esaú Bojórquez-Velázquez, José P. Martínez, Antonio De León-Rodríguez, Eduardo Espitia-Rangel, Alfredo Herrera-Estrella

**Affiliations:** 1 IPICYT, Instituto Potosino de Investigación Científica y Tecnológica A.C., Camino a La Presa San José 2055, Lomas 4ª Sección, 78216 San Luis Potosi, SLP México; 2 Unidad de Genómica Avanzada, Cinvestav, CP 36824 Irapuato, Guanajuato Mexico; 3 Red de Estudios Moleculares Avanzados, INECOL, Campus III, 91073 Xalapa, Veracruz México; 4 INIFAP, Instituto Nacional de Investigaciones Forestales Agrícolas y Pecuarias, 56250 Texcoco, Estado de México México

**Keywords:** Amaranth, GC–MS, Metabolite profile, Phosphonate metabolism, Salt stress, Transcriptomics, Water deficit, Wild species

## Abstract

**Main conclusion:**

*A. hybridus* tolerance to salinity depends on constitutively active mechanisms, whereas *A. hypochondriacus* tolerance to salt and water deficit depends on a constitutive protection and a robust transcriptional response.

**Abstract:**

Drought and soil salinity are two environmental factors that significantly affect crop production. To gain a better understanding of how amaranth responds to these abiotic stresses, we analyzed the transcriptomic and metabolomic changes in the leaves of *Amaranthus hybridus*, a wild species, and *A. hypochondriacus*, a cultivated species used for seed production. We identified differentially expressed genes (DEGs) between the two species and under different stress conditions. Control plants of *A. hypochondriacus* exhibited higher expression levels of genes associated with photosynthesis, amino acid metabolism, fatty acid metabolism, sulfur metabolism, thiamine metabolism, and secondary metabolism. Notably, *A. hybridus* under salt stress showed an up-regulation of genes related to phosphonate and phosphinate metabolism and steroid biosynthesis. In contrast, the response of *A. hypochondriacus* to salt stress was characterized by increased expression of ABC transporters and genes involved in fructose, mannose, trehalose, porphyrin, thiamine, and monoterpenoid metabolism. When subjected to both types of stresses, *A. hypochondriacus* showed up-regulation of MAPK signaling pathways, ABC transporters, galactose, branched-chain amino acid (BCAA) degradation, and the production of defense compounds. Both amaranth species modulated their metabolic processes in response to drought and salinity stress towards cell wall modification, as well as the metabolism of pectin and lignin, while also producing antimicrobial and antifungal metabolites. Additionally, we detected differential accumulation of compounds, including methylphosphonate, 2-hydroxyethylphosphonate, and several metabolites related to fatty acid metabolism in the leaves of both amaranth species.

**Supplementary Information:**

The online version contains supplementary material available at 10.1007/s00425-026-04927-x.

## Introduction

Sustainable agriculture faces several challenges in producing sufficient food to meet the needs of the growing global population, especially when it comes to providing healthy options. Additionally, food production is significantly threatened by unpredictable global climate changes (Fanzo et al. 2021). Factors, such as drought and soil salinity, greatly affect crop yields. In this context, plants that are tolerant to these stresses employ various physiological and molecular responses to survive such adverse conditions. Wild relatives of crops, which have withstood numerous climate changes, serve as valuable sources of genetic traits linked to abiotic stress tolerance. Therefore, understanding the molecular responses of these tolerant plants is essential for developing breeding strategies aimed at sustainable food production (Abdelrahman et al. 2018; Saharan et al. 2022).

Various strategies of the plants have been developed in response to stress which includes the accumulation of osmo-protectants (proline, sugars, and glycine betaine). These osmolytes can trigger plant stress tolerance mechanisms, such as the activation of resistance genes, the increased levels of enzymatic and non-enzymatic antioxidants, the enhanced cell membrane integrity, and the protection of the photosynthetic apparatus, especially the photosystem II (PSII) complex (Mahajan and Tuteja 2005). Moreover, plant plasma membranes (PMs) act as biological barriers, safeguarding cell contents and organelles from environmental stresses while also generating highly regulated signaling outputs (Hou et al. 2016). In this regard, changes in membrane lipids can directly influence the properties of membrane proteins and activate signal transduction pathways (Hou et al. 2016; Hafeez et al. 2023).

The Amaranthaceae family includes several salt-tolerant species, and some plants from the *Amaranthus* genus are consumed as vegetables, such as *A. hybridus*. Others, like *A. hypochondriacus* L., are utilized for grain production. *A. hypochondriacus* L., which is native to Mexico, was domesticated from at least two wild ancestors: *A. hybridus* and *A. powellii.* These ancestors thrive in harsh conditions; *A. hybridus* is adapted to saline soils, while *A. powellii* can grow under low water conditions in Mexican territories (Espitia-Rangel et al. 2010).

There is a growing interest in amaranth plants due to their remarkable ability to adapt to abiotic stress conditions, such as drought and salinity (Huerta-Ocampo et al. 2014). Furthermore, amaranth grains have a higher protein content than traditional cereals and possess a well-balanced profile of essential amino acids and bioactive peptides (Bojórquez-Velázquez et al. 2019). Amaranth seeds also contain important phytochemicals, such as rutin and nicotiflorin, which exhibit high antioxidant activity (Barba de la Rosa et al. 2009). Despite the evidence highlighting amaranth as a promising species for producing high-quality food in semiarid and arid regions where other crops struggle to grow, little attention is paid to its cultivated varieties and even less to its wild relatives. Gaining a better understanding of the mechanisms that enable amaranth to adapt to climate conditions could inform strategies for developing highly productive crops (Estrada et al. 2021).

In this study, we examined the leaf transcriptome responses of amaranth to water deficit and salt stress, comparing the arable *A. hypochondriacus* with its highly salt-tolerant ancestor, *A. hybridus*. We classified differentially expressed genes (DEGs) as specific to the species or as responses to water deficit or salinity. Additionally, we analyzed the leaf metabolite profile using GC–MS. Our results provide insights into how amaranth species respond to and adapt to abiotic stresses.

## Materials and methods

### Plant material, growth conditions, and stress treatments

*Amaranthus hypochondriacus* cv. Nutrisol and *Amaranthus hybridus* lines were obtained from the National Institute of Forestry, Agricultural and Livestock Research (INIFAP), Texcoco, Mexico. The seeds were placed in a sterile wet substrate (BM2 Germination Mix; Berger, Saint Modeste, QC, Canada) and kept at 4 °C for 12 h. Subsequently, they were transferred to a growth chamber set at 25 °C and subjected to 12 h light/dark cycles. After 11 days in the growth chamber, the seedlings were moved to a greenhouse and planted in plastic pots that were 10 cm in diameter and 20 cm high. For each of the three experimental groups (control, water deficit, and salinity), 12 to 15 plants per species were placed. The plants were watered every third day for 19 days after entering the greenhouse until they were one month old at which point the treatments began. The stress treatments were implemented using a randomized complete block design. Control plants were watered, as mentioned above, throughout the experiment. For saline stress, 100 ml of 0.15 M NaCl was applied daily for 7 days, followed by 100 ml of 0.3 M NaCl for the next 4 days, and 100 ml of 0.5 M NaCl for the final 3 days. Plants subjected to water stress were kept without irrigation for 14 days during the treatment period. Plants showing delayed development, even before treatment, were excluded from the analysis. From three plants per treatment group (3 control, 3 salt stress, 3 drought stress), we collected mature, non-senescent leaves (specifically leaves 5, 6, and 7 from the apex of each plant) and pooled them. This constituted one replicate, and the experiment included three biological replicates. All samples were frozen in liquid nitrogen and stored at −80 °C.

### Sugars, phenolic compounds, flavonoids, and chlorophyll measurements

To extract total sugars, dried leaf samples (30 mg) were subjected to extraction with 1 ml of 80% methanol solution for 15 min at 80 °C. The samples were then centrifuged at 5000 g for 10 min, and the resulting pellet was washed sequentially with 0.5 ml of 50% methanol, 0.5 ml of 20% methanol, and water. All recovered supernatants were combined, concentrated using a speed vacuum drier, and re-suspended in 20% ethanol. An aliquot of 2 µl was then injected into an HPLC system (Agilent Technologies, Santa Clara, CA, USA) equipped with a SUGAR SP0810 column (Shodex, Showa Denko America Inc., NY, USA) at 80 °C, with a refractive index detector (RID) set to 50 °C. The mobile phase consisted of distilled water flowing at a rate of 0.3 ml/min. Glucose and sucrose standards were used to quantify the levels of glucose and sucrose in the samples.

To quantify total phenolic compounds, 20 µl of a methanolic extract was mixed with 780 µl of Milli-Q water, 50 µl of Folin–Ciocalteu reagent, and 150 µl of 20% N_2_CO_3_. The samples were incubated in the dark for two hours. A standard curve using gallic acid (GA) was employed to quantify the phenolic content in the samples. The absorbance of the samples was measured at 765 nm using a Multiskan microplate spectrophotometer (Thermo Fisher Scientific, Waltham, MA, USA). The results were reported as micrograms of gallic acid equivalents (GAE) per milligram on a dry weight basis (DW).

To quantify flavonoids, 200 µl of a methanolic extract was mixed with 800 µl of a 2% AlCl_3_ solution. The samples were then incubated in the dark for 10 min. A quercetin (Q) standard curve was prepared to determine the flavonoid content in the samples. The absorbance of the samples was measured at 430 nm using a Multiskan microplate spectrophotometer (Thermo Fisher Scientific). The results were reported as micrograms of Quercetin Equivalents (QE) per milligram of dry weight (DW).

For chlorophyll quantification, dried leaf samples (15 mg) were mixed with 1 ml of absolute methanol at room temperature. The mixture was agitated at 800 rpm in a ThermoMixer (Eppendorf AG, Hamburg, Germany) for 15 min in the dark. Afterward, the samples were centrifuged at 17,000 g for 10 min, and the supernatants collected in a clean tube. This extraction process was repeated two more times, and the supernatants were collected. As described by Warren et al. (2008), the absorbance of 200 μl of the extract was measured at 652 and 665 nm, and the reading corrected by dividing by 0.51. Chlorophyll a and b content was calculated using the following Eqs. (1 and 2):

1 $$Chlorophyll\,a\,\left( {\mu g/mg} \right) \, = \, \left( { - 8.096A_{652} + \, 16.517A_{665} } \right)/sample\,dry\,weight$$

2 $$Chlorophyll\,b\,\left( {\mu g/mg} \right) \, = \, \left( {27.44A_{652} {-} \, 12.167A_{665} } \right)/sample\,dry\,weight$$

### Identification of volatile and semi-volatile compounds by gas chromatography

The methanolic extracts of amaranth leaves were analyzed by GC–MS using a 7820A/5977E System (Agilent Technologies) equipped with a Zebron ZB5-MS column (30 m × 0.25 mm × 0.25 mm) from Phenomenex (Torrance, CA, USA). The operating conditions included injector and detector temperatures set at 250 °C and 280 °C, respectively. The temperature program began with a 10 min hold at 60 °C, followed by an increase of 3 °C per minute until it reached 240 °C, where it was maintained for an additional 10 min. Helium was used as the carrier gas at a flow rate of 1 ml/min, and the mass range analyzed was 40–400 m/z, with a scan rate of 1.0 s/s for sample analysis. The injector was operated in split-less mode at 250 °C. The mass spectrometer detector functioned in Electron Impact Ionization mode at 70 eV, utilizing both SIM and Scan modes. Compounds were identified by comparing the obtained spectra with the reference spectra in the NIST library database provided by the equipment.

### RNA sequencing, transcriptome assembly, and unigene functional annotation

Total RNA was extracted from three biological replicates of leaves from stressed (salinity or water deficit) and non-stressed (watered) plants using the TRIzol protocol specified by the manufacturer (Thermo Fisher Scientific). TruSeq libraries for all samples were sequenced on the Illumina NextSeq 500 1 × 75 high at the Advanced Genomics Unit facilities. Raw RNA-Seq data are available under Gene Expression Omnibus (GEO) accession number: GSE288330. The overrepresented sequences and the quality of the raw reads were determined using the FASTQC program (Andrews 2010). Low-quality reads (QC < 20), overrepresented sequences, and Illumina adapters were removed using the ILLUMINACLIP option in Trimmomatic (Bolger et al. 2014). Also, low-quality bases from the beginning or from the end (LEADING:15, TRAILING:15) were removed and reads longer than 30 bp (MINLEN:30) were conserved for further analysis.

Transcriptome assemblies of *A. hybridus* and *A. hypochondriacus* were obtained using TRINITY v2.1.1 (Haas et al. 2013) with the options "–normalize_reads" and "–trimmomatic", which are integrated into the TRINITY program. Transcript redundancy from Trinity "genes" was filtered out using CD-HIT (Li and Godzik 2006) at 90% sequence identity. Transcriptome quality was evaluated with BUSCO v2.0 using the eukaryote09 database (Simão et al. 2015).

The unigenes were compared using BLAST against the NCBI NR database (BLASTX, e value < 1e-^5^) for GO functional annotation of transcripts from *A. hybridus* or *A. hypochondriacus.* The BLAST results were loaded into the Java version of the blast2GO v2.3.5 program and mapped to the Gene Ontology database (Conesa and Götz 2008).

### DEG analysis of salinity and water-deficit treatments

High-quality reads of each library were mapped to the transcriptome of *A. hybridus* or *A. hypochondriacus* using the preset options “-very-sensitive” in the “-end-to-end” mode of BOWTIE2 v2.3.0 (Langmead and Salzberg 2012). An in-house bash script was used to count unique reads from the SAM files. Briefly, the command awk was used to get reads with FLAG alignments of 0 or 16. The reads were then removed with suboptimal alignment scores to obtain only reads with unique alignments to the reference (awk '{if ($2 =  = 0 || $2 =  = 16) print $0}' Ahyb_R1.trimm.sam | grep -v 'XS:i' |cut -f 3 |sort |uniq -c > RNAseq_R1_Uniq.tx t). A table count reads matrix was constructed in an R environment and used to identify DEGs with the edgeR package (Robinson et al. 2010). Pairwise analysis of stressed (water-deficit or salinity) plant samples vs control plant samples was carried out using the exact test of the edgeR package and filter parameters of FDR < 0.01 and log2FC > 2 or log2FC < −2.

Orthologs between *A. hybridus* and *A. hypochondriacus* were identified using OrthoFinder (Emms and Kelly 2019) with default parameters. Additionally, the proteomes of *A. thaliana* and *A. hypochondriacus* were sourced from Phytozome. To visualize the orthologs between the species under both stress and control conditions, transcripts per million (TPM) were used, and the "scale = row" option from the pheatmap package in R was applied for plotting.

### GO enrichment analysis of the DEG from salt and water-deficit stress

The DEGs from *A. hybridus* and *A. hypochondriacus* were categorized into up-regulated and down-regulated groups based on the type of stress—either water deficit or salinity. The topGO package (Alexa et al. 2006) was utilized to identify GO-enriched terms for the genes in both the up- and down-regulated groups, as well as for genes regulated specifically under salinity or water deficit in each *Amaranthus* species. GO terms with a *P* value of ≤ 0.05 and containing more than three genes in each category were considered to be overrepresented. To minimize redundancy among GO terms, the REVIGO tool (http://revigo.irb.hr) was used with the tiny option to search for similarity.

## Results

### *A. hypochondriacus* is highly tolerant to water-deficit and salt stress

**Fig. 1:**
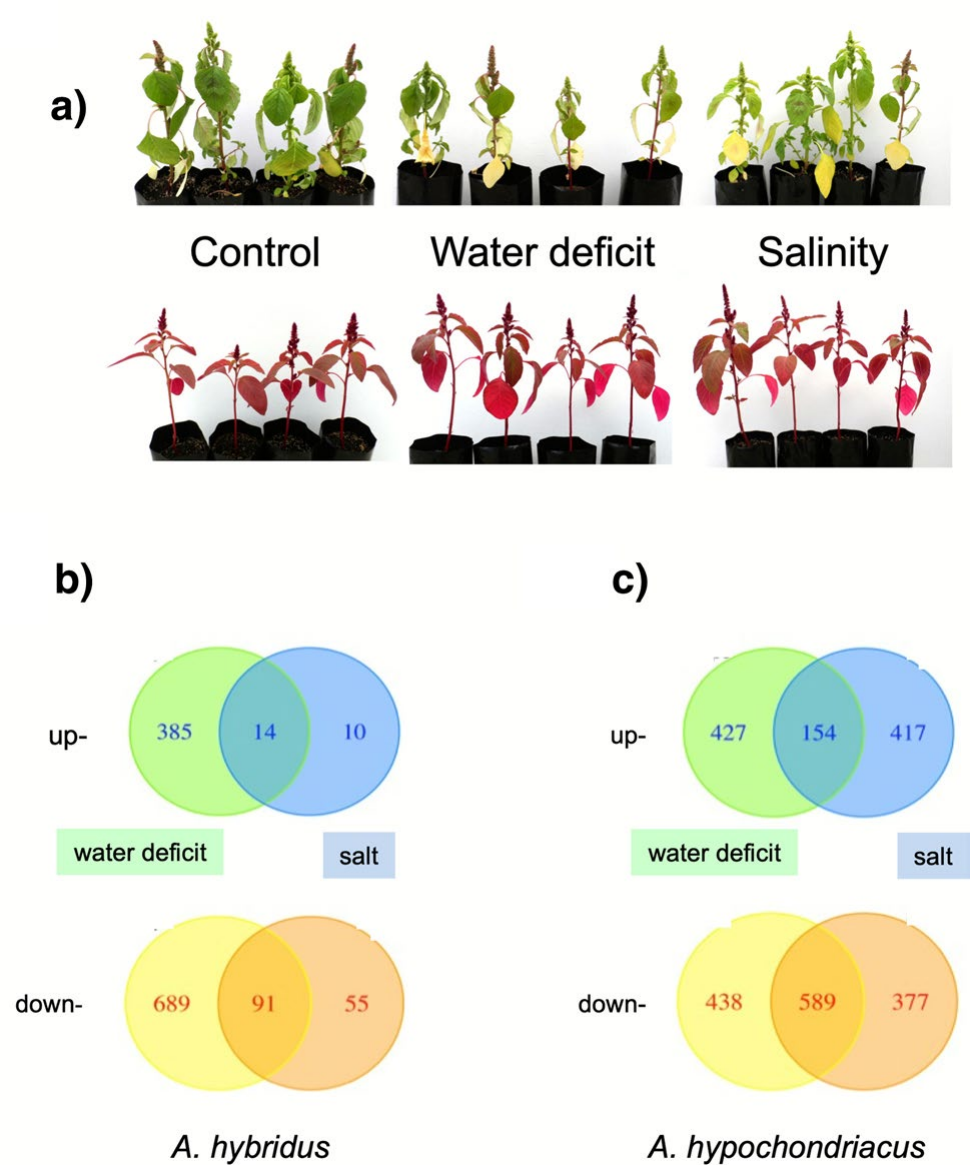
Response of the *Amaranthus* species to stress. **A** Phenotypic response. The photographs show representative plants of *A. hybridus* (top panels) and *A. hypochondriacus* (bottom panels) under the indicated conditions. **B** and **C** Transcriptomic response. Venn diagrams of up- and down-regulated genes when plants were subjected to water deficit or salt stress for *A. hybridus* (**B**) and for *A. hypochondriacus* (**C**)

To our surprise, amaranths were able to grow under saline conditions where other cereals cannot. We gradually increased the salt concentration to determine the conditions under which amaranths respond. When we applied 0.3 M and 0.5 M concentrations of NaCl, we noticed effects on the phenotype, indicating the high salinity tolerance of amaranths. Under control conditions, *A. hybridus*, a wild species, produces a high biomass; however, under stress, this biomass decreases significantly. Older leaves displayed signs of chlorosis, which were more pronounced in plants subjected to water deficit than those under salt stress (Fig. 1A). In contrast, *A. hypochondriacus*, an arable species, did not exhibit significant changes when subjected to water deficit or salt stress compared to those grown under control conditions (Fig. 1A). Despite the stress, all plants maintained their turgor and reached the reproductive stage without a time lag.

### Amaranth species show contrasting transcriptome responses to high salinity

We obtained RNA sequencing libraries from the leaves of control plants, as well as plants subjected to water deficit and salt stress, from two species of amaranth. The sequencing of 18 RNA libraries produced on average 19.71 million raw reads per library (Fig. S1). The RNA sequencing libraries from *A. hybridus* and *A. hypochondriacus* were assembled using the Trinity software. Specifically, 204,094,843 filtered reads were utilized for *A. hybridus*, resulting in the assembly of 30,156 non-redundant genes. For *A. hypochondriacus*, 150,482,640 filtered reads were used, yielding 30,946 non-redundant genes (refer to Table S1). The quality of the assemblies was assessed using BUSCO with the eukaryotic database, which indicated a 63% completeness for *A. hybridus* and 65.1% for *A. hypochondriacus* (Table S1). These assemblies were used for further analyses.

We conducted pairwise comparisons of stress versus control leaves for both species. We used high-quality reads that uniquely mapped to the corresponding transcriptome assembly to identify DEGs. These DEGs were organized in Venn diagrams based on the conditions under which they were identified (Fig. 1B). Interestingly, *A. hybridus* showed fewer DEGs under high salinity stress (170) compared to water-deficit conditions (1179). Among these, 14 up-regulated and 91 down-regulated DEGs were common to both water deficit and salt stress. In contrast**,** *A. hypochondriacus* showed a similar number of DEGs under salinity (1537) and water deficit (1608) conditions, with 154 up-regulated and 589 down-regulated DEGs shared between the two stresses (Fig. 1B; Supplementary Files S1 and S2).

As mentioned above, *A. hybridus* exhibited signs of damage when experiencing water deficit, but not under salt stress. This observation aligns with the transcriptome results and supports the idea that *A. hybridus* is naturally adapted to thrive in saline soils. While *A. hypochondriacus* did not display any visible signs of stress, it responded strongly at the transcriptional level to both salt stress and water deficit.

### Species-specific transcriptional responses

**Fig. 2:**
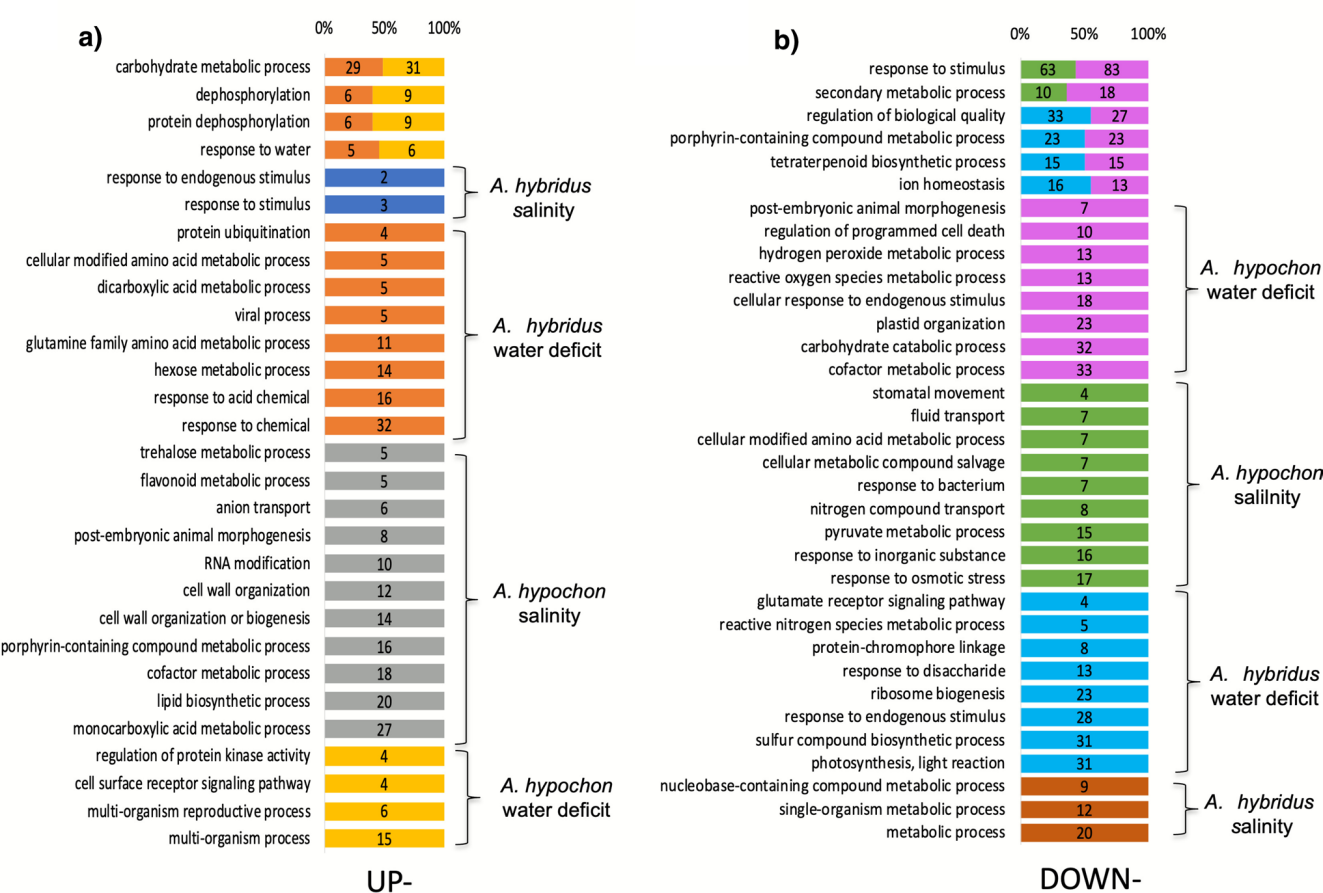
GO term enrichments of differentially expressed genes in response to stress. GO terms enriched in genes up-regulated (**A**) and down-regulated (**B**) in *A. hybridus* and *A. hypochondriacus* when subjected to water deficit or salt stress

We categorized DEGs into species- and response-specific groups based on the transcriptome assemblies for *A. hybridus* and *A. hypochondriacus*. GO enrichments derived from the DEGs using topGO (*P* < 0.05) revealed distinct groups of up- and down-regulated genes in response to water deficit and salinity (see Figs. 2 and S2). Under salt stress, *A. hybridus* exhibited up-regulated gene groups associated with responses to stimuli, while metabolic processes were down-regulated. In conditions of water deficit, this species showed up-regulation of genes involved in ubiquitination, dicarboxylic acid metabolism, glutamine metabolism, and hexose metabolism. Conversely, the down-regulated genes were linked to glutamate receptors, reactive nitrogen species, sulfur compound metabolic process, ribosome biogenesis, and the photosynthetic light reaction.

In *A. hypochondriacus*, a specific set of up-regulated genes under salinity stress was associated with processes related to trehalose and flavonoid metabolism, anion transport, RNA modification, and cell wall organization or biogenesis. Additionally, these genes were linked to porphyrin-containing compound and cofactor metabolism, as well as lipid biosynthesis and monocarboxylic acid metabolism. Conversely, the down-regulated genes were related to stomatal movement, fluid transport, responses to bacteria, and the pyruvate metabolic process. Under conditions of water deficit, we observed up-regulation of genes associated with kinase activity and cell surface receptors. In contrast, down-regulated genes were connected to plastid organization, reactive oxygen species (ROS) and hydrogen peroxide metabolism, programmed cell death, and carbohydrate catabolism (see Fig. 2).

**Fig. 3:**
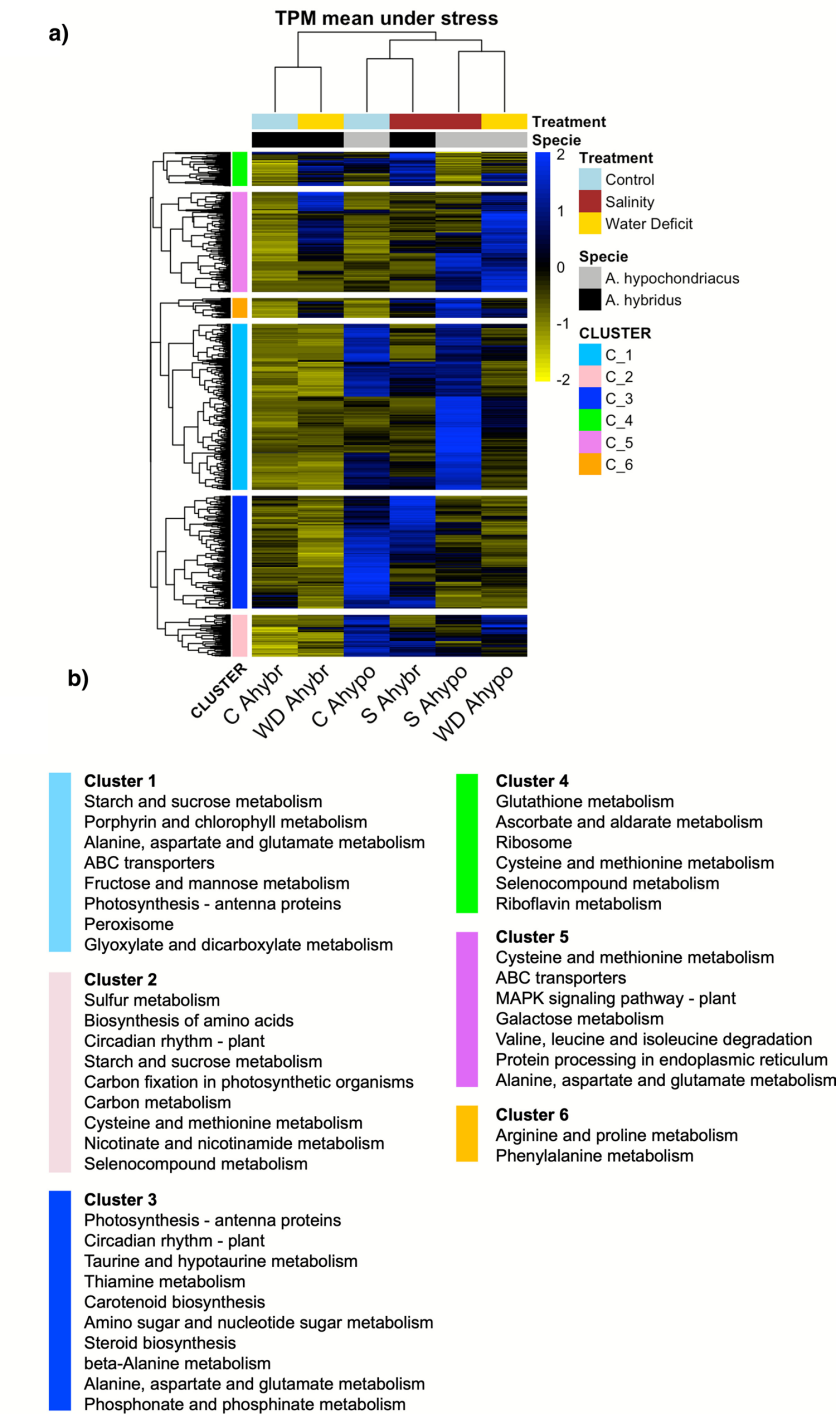
Expression profile of *A. hybridus* and *A. hypochondriacus* orthologs. **A** Heat map shows the comparison of the Z-scores for transcripts per million (TPM) of orthologs from both species under control conditions and under water deficit or salt stress. TPM values were Z-score transformed using the pheatmap package ("scaled = row") in R. **B** GO terms enriched in the main clusters shown in **A**

We identified the orthologs of *A. hybridus*, *A. hypochondriacus*, and *Arabidopsis thaliana* using OrthoFinder and the genome of *A. hypochondriacus* (Supplementary File S3). The expression levels of DEGs orthologous to *A. hybridus* and *A. hypochondriacus*, normalized to transcripts per million (TPM), are displayed in Fig. 3A. Hierarchical clustering, based on the Euclidean distance method, grouped the DEGs into species-specific clusters that exhibited correlated expression patterns.

Cluster 1 consists of two sub-clusters that group DEGs showing high expression levels in *A. hypochondriacus* under both control conditions and increased expression levels under salt stress (Fig. 3A). These DEGs are associated with the biosynthesis of secondary metabolites, starch and sucrose metabolism, porphyrin and chlorophyll metabolism, amino acid metabolism (specifically alanine, aspartate, and glutamate), as well as sugar metabolism (fructose and mannose). Additionally, this cluster includes genes related to photosynthesis, peroxisome metabolism, glyoxylate and dicarboxylate metabolism, the ABC14 gene involved in stomatal aperture regulation, and ABC13, which plays a key role in auxin homeostasis (Fig. 3B).

Cluster 2 encompasses genes that are highly expressed in *A. hypochondriacus* under control conditions and during water deficit (Fig. 3A). This cluster primarily consists of genes enriched in functions related to sulfur metabolism, carbon fixation, carbon metabolism, and the metabolism of nicotinate and nicotinamide, as well as cysteine, methionine, and selenium compounds (Fig. 3B).

Cluster 3 is divided into two sub-clusters, highlighting the most notable differences between the two species, *A. hypochondriacus* and *A. hybridus*, particularly under salt stress conditions (Fig. 3A; Supplementary File S3). The first subcluster features the genes of *A. hypochondriacus* that are highly expressed, which are enriched in various functions including photosynthesis, metabolism of amino sugars and nucleotide sugars, thiamine and porphyrin metabolism, chlorophyll metabolism, biosynthesis of secondary metabolites, circadian rhythm, and glycolysis/gluconeogenesis. The second sub-cluster consists of genes from *A. hybridus* that are highly expressed under salt stress. These genes relate to several metabolic pathways, including taurine and hypotaurine metabolism, steroid biosynthesis, cyanoamino acid metabolism, general amino acid metabolism (specifically for alanine, aspartate, glutamate, beta-alanine, glycine, serine, and threonine), phosphonate and phosphinate metabolism, linoleic acid metabolism, and glyoxylate and dicarboxylate metabolism, as well as glycerophospholipid metabolism (Fig. S3).

Cluster 4 represents a relatively small set of genes that exhibit high levels of expression in *A. hybridus* under water deficit and salt stress (Fig. 3A). This cluster is characterized by its enrichment in genes involved in glutathione, ascorbate, riboflavin, and aldarate metabolism, cysteine, methionine, and seleno-compound metabolism, and ribosomes (Fig. 3B).

Cluster 5 is divided into two sub-clusters. The first sub-cluster encompasses genes that show high expression levels under both stresses in *A. hypochondriacus* (Fig. 3A and Supplementary File S3)*.* This sub-cluster is enriched in DEGs related to ABC transporters and MAPK signaling pathways. The second sub-cluster includes genes that are highly expressed in both species under water deficit conditions. This subcluster encompasses genes involved in galactose metabolism, the degradation of amino acids (specifically valine, leucine, and isoleucine), and protein processing in the endoplasmic reticulum (Fig. 3B and Supplementary File S3).

Genes in cluster 6 represent a salt stress specific responsive gene set for *A. hypochondriacus* (Fig. 3A). These genes are related to arginine, proline, and phenylalanine metabolism (Fig. 3B).

### Sugars, phenolic compounds, and chlorophyll contents undergo significant changes in amaranth leaves in response to abiotic stress

**Fig. 4:**
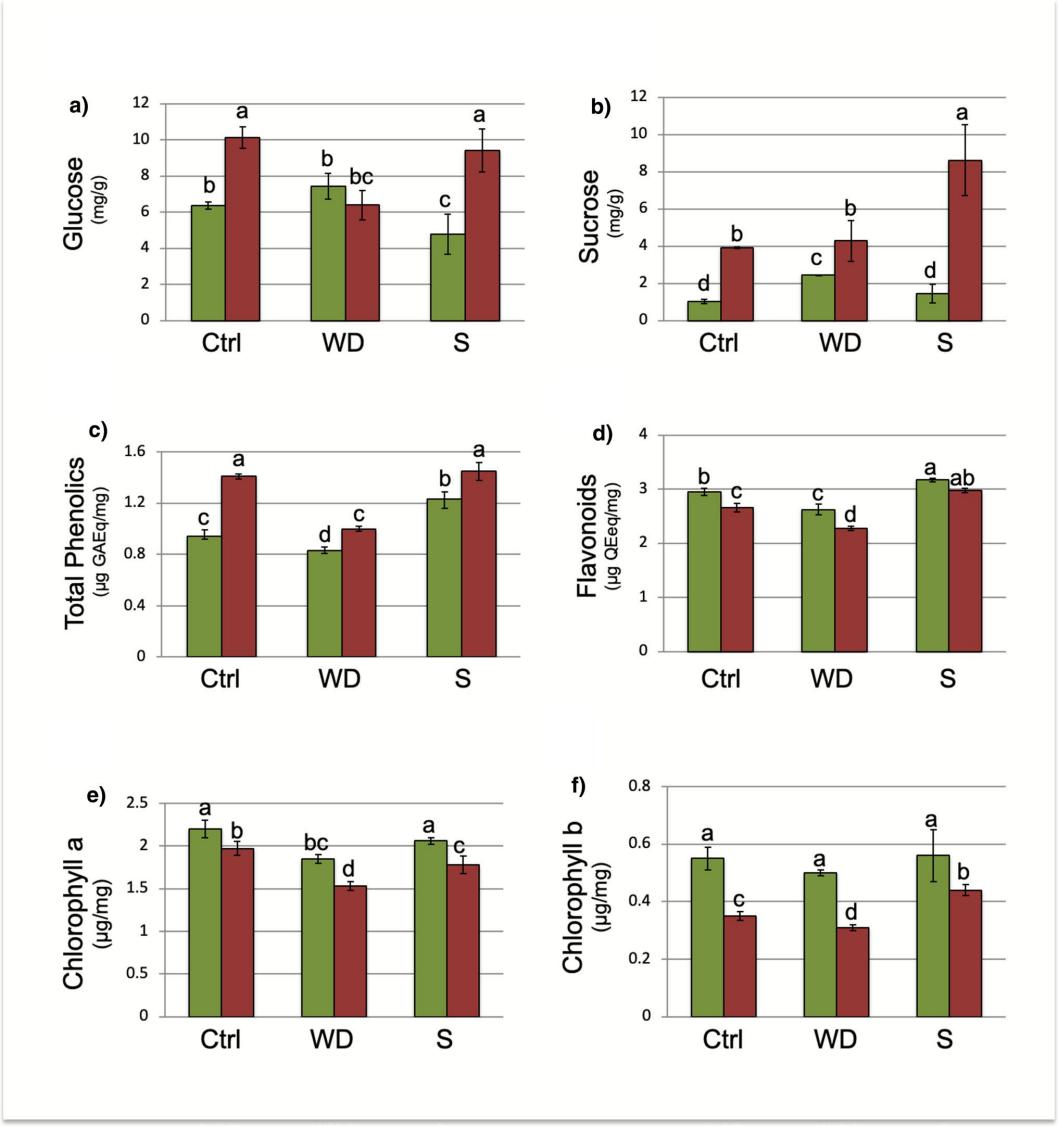
Endogenous content of key metabolites in amaranth leaves under stress conditions. Green bars, *A. hybridus*; Red bars, *A. hypochondriacus.* Glucose (**A**) and sucrose (**B**) in mg/g dry weight. **C** Phenolic compounds as µg Gallic Acid Equivalents/mg dry weight (GAEq). **D** Flavonoids as µg Quercetin Equivalents/mg dry weight (QEq/mg). **E**, **F** Chlorophyll a (**E**) and chlorophyll b (**B**) in µg/mg dry weight. Ctrl, control conditions; S, salinity stress; WD, water deficit. Bars show the mean values ± SD (*n* = 3), and the same letters above the columns indicate no significant differences at *P* ≤ 0.05

Under control conditions, glucose accumulation was higher in *A. hypochondriacus* (Fig. 4A, red bars) than *in A. hybridus* (Fig. 4A, green bars)*.* However, under water deficit, glucose levels decreased in *A. hypochondriacus* to a level similar to that of *A. hybridus* under control and water deficit conditions. In response to salt stress, glucose levels decreased in *A. hybridus*, while *A. hypochondriacus* showed no significant changes compared to control conditions (Fig. 4A).

Regarding sucrose accumulation, no significant changes were observed in *A. hypochondriacus* when comparing control to water deficit conditions. However, there was a substantial accumulation of sucrose noted under salt stress (Fig. 4B). Under control conditions, phenolic compounds were higher in *A. hypochondriacus* than in *A. hybridus*. Both species experienced a decrease in phenolic content under water deficit conditions. Notably, *A. hybridus* exhibited a significant increase in phenolic compounds when subjected to salt stress (Fig. 4C).

In terms of flavonoid contents, *A. hybridus* consistently had higher levels than *A. hypochondriacus*. Flavonoid contents decreased under water deficit conditions but increased under salt stress (Fig. 4D). Additionally, in all treatments, the amounts of chlorophyll a (Fig. 4E) and chlorophyll b (Fig. 4F) were greater in *A. hybridus* than in *A. hypochondriacus*, with both types of chlorophyll showing a decline when the plants were subjected to stress.

### Changes in the accumulation of volatile and semi-volatile compounds between species and the stress applied

**Fig. 5:**
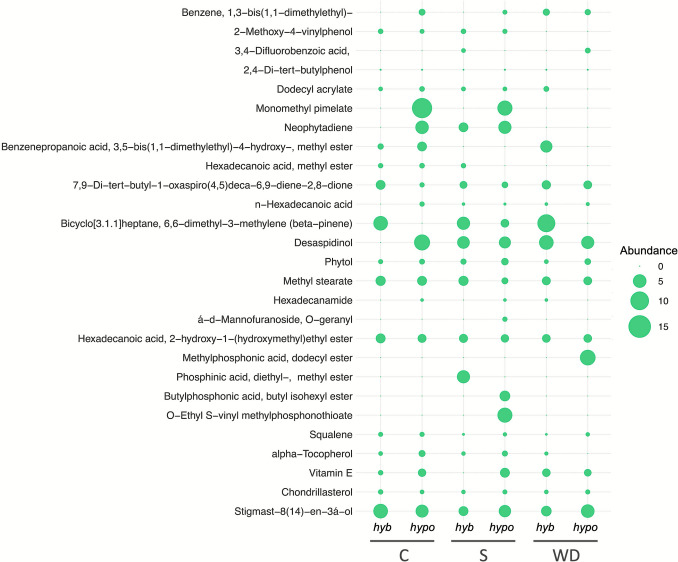
Dot-map of the relative abundances of selected compounds identified by GC–MS in amaranth leaf. *hyb*, *Amaranthus hybridus*; *hypo*, *Amaranthus hypochondriacus*; C, control; S, salinity stress; WD, water deficit

To gain a better understanding of how amaranth responds to stressful conditions, we conducted non-targeted metabolomic analyses to explore the metabolic changes occurring in the plants. This approach allows us to identify new compounds although it does not enable quantification since no standards were used. We observed changes in the relative abundance of volatile and semi-volatile compounds between species and when the plants were subjected to water deficit or salt stress (Fig. 5).

Several compounds related to fatty acid metabolism were detected, among them palmitic acid (*N*-hexadecanoic acid) and its derivatives. Hydrocarbons, including squalene, α-tocopherol, and chondrillasterol, were identified in all treatments. We also detected differential accumulation of organic compounds and those related to antibacterial, antifungal, and anthelmintic functions, including desaspidinol, 17,9-Di-tert-butyl-1-oxaspiro(4,5)deca-6,9-diene-2,8-dione, and the terpene glycoside α-D-mannofuranoside-*O*-geranyl (Fig. 5; Table S2).

Phytol and neophytadiene (diterpenoids) were detected, with phytol found in all samples. Neophytadiene, a product of phytol degradation, was detected in *A. hypochondriacus* control and salt-stressed plants, as well as in *A. hybridus* under salt stress. Additionally, monomethyl pimelate was found in high abundance in *A. hypochondriacus* under both control and salt-stress conditions. We detected compounds related to phosphonate metabolism, such as methylphosphonic acid and *O*-ethyl-vinyl methylphosphonothioate, in *A. hypochondriacus* under salinity and water deficit, respectively. Phosphinic acid and butyl phosphonic acid were detected only in *A. hybridus* under salt stress.

## Discussion

Drought and soil salinity represent significant agricultural threats, further exacerbated by climate change (Shivaraj et al. 2021). The genus *Amaranthus* includes wild species like *A. hybridus*, which is regarded as one of the direct ancestors of the more commonly cultivated species, *A. hypochondriacus*. Both species were capable of growing at NaCl concentrations of up to 500 mM. When plants can thrive under such salt concentrations, they are classified as halophytes (Hameed et al. 2024). Phenotypically, *A. hybridus* is more susceptible to water deficit than *A. hypochondriacus*, as evidenced by reduced biomass production and stunted plants displaying signs of chlorosis. At the transcriptional level, *A. hybridus* showed minimal changes in leaf gene expression under salt stress, consistent with its natural habitat in highly saline soils. In contrast, although *A. hypochondriacus* exhibited few phenotypic changes under water and salt stress, it demonstrated a more robust transcriptional response compared to *A. hybridus*.

### *A. hypochondriacus* under control conditions showed high levels of expression of genes related to photosynthesis and sulfur metabolism that protect against stress

Under control conditions, *A. hypochondriacus* showed higher expression levels of genes related to photosynthesis, and sulfur, seleno-compound, and carbohydrate metabolism. Several isoforms of glucose-6-phosphate isomerase and glyceraldehyde-3-phosphate dehydrogenase, which are related to glycolysis and the Calvin cycle, were identified.

**Fig. 6:**
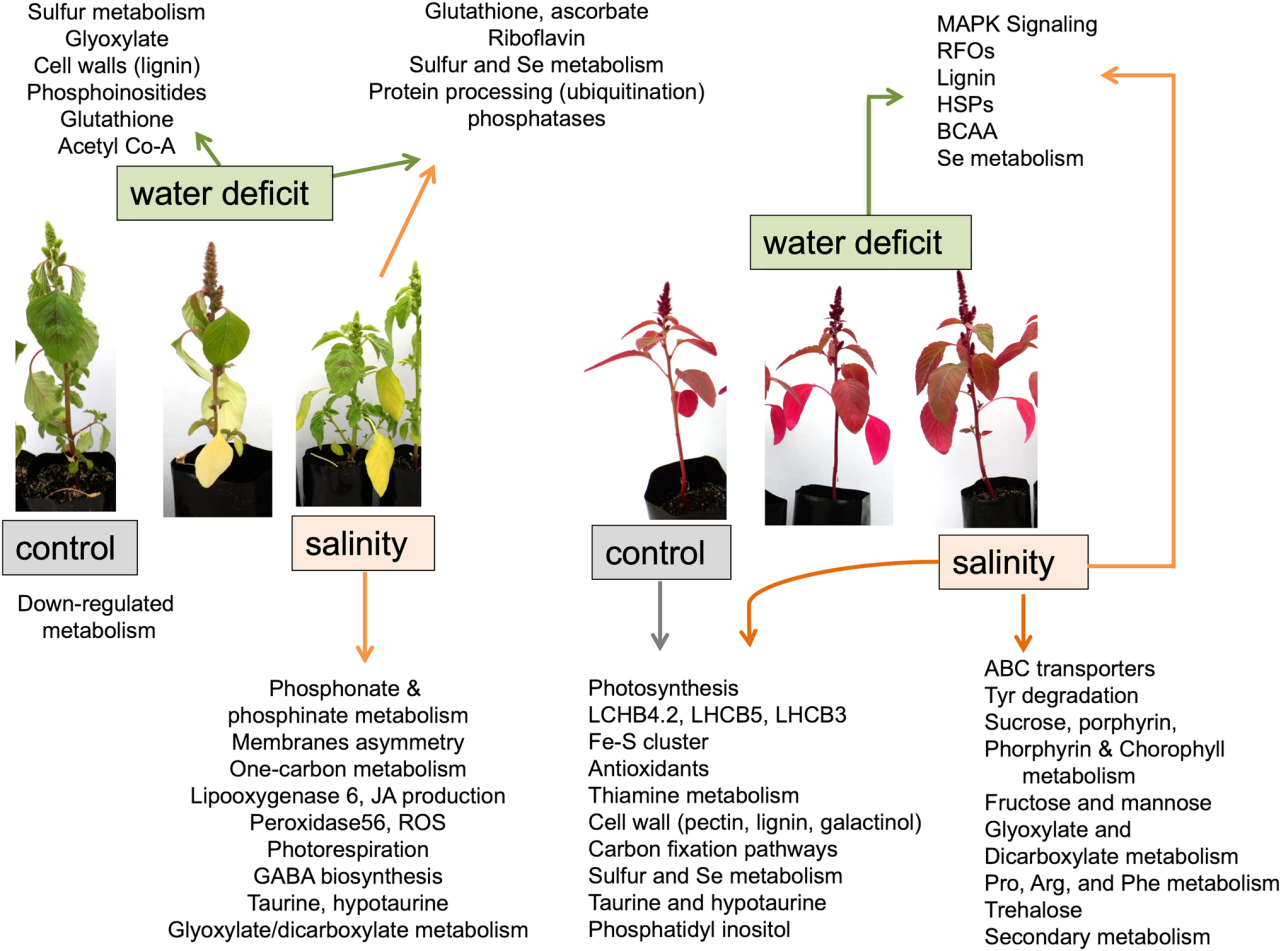
Schematic diagram representing the main amaranth responses to water deficit and salinity stress. *A. hybridus*, the wild species, has green leaves and, under salt stress, showed DEGs related to phosphonate and phosphinate metabolism, membrane protection by regulating asymmetry, protection against harmful ROS via peroxidases, and taurine and hypotaurine metabolism. Under salt stress, *A. hybridus* showed increased phosphatases involved in phosphonate and phosphinate degradation, RFOs, and ubiquitination of proteins for their degradation. *A. hypochondriacus*, the cultivated species, has red leaves. Under control conditions, it shows high expression levels of genes related to photosynthesis and chloroplast protection, which enhances carbon fixation. This species also shows increased metabolism of sulfur and thiamine and greater production of antioxidants. The modifications in the cell wall involve the regulation of pectin and lignin biosynthesis. Furthermore, it exhibits increased production of gamma-aminobutyric acid (GABA) and engagement in one-carbon metabolism. In *A. hypochondriacus*, the rapid response and adaptability to water deficit and salinity stress could be mediated by activation of MAPK signaling, production of defense compounds, RFOs, and BCAA metabolism, as well as HSPs

Our transcriptome analyses indicate that *A. hypochondriacus* shows elevated expression of transcripts linked to photosynthesis. Specifically, light-harvesting complexes, such as CP29.2 (LHCB4.2), CP26 (LHCB5), and LHCB3, were up-regulated. Chlorosis, a common symptom of iron (Fe) deficiency resulting from impaired chlorophyll biosynthesis, is typically associated with decreased photosynthetic rates. However, *A. hypochondriacus* did not display chlorosis in its leaves (Fig. 1). These data agree with the measurements of photosynthetic parameters showing that *A. hypochondriacus* has a better response to stress than *A. hybridus* (Vargas-Ortiz et al. 2021) (Fig. 6).

We observed high expression levels of transcripts encoding enzymes involved in chlorophyll metabolism, including chlorophyllide a oxygenase and magnesium–protoporphyrin IX monomethyl ester cyclase; both of these enzymes are dependent on Fe-S clusters (Tran et al. 2023). Chlorophyllide a oxygenase catalyzes the conversion of chlorophyll a to chlorophyll b, while increases in chlorophyll b can enhance photosynthetic electron transport and carbon assimilation rates (Biswal et al. 2024). Mg-chelatase is a key component of chlorophyll biosynthesis and plastid signaling, modulating porphyrin biosynthesis and alleviating Fe deficiency-induced stress (Tran et al. 2023). Chloroplastic adenylate kinase 2 plays a key role in ADP synthesis within chloroplasts, which is important for CO_2_ fixation, growth, and plant responses. This gene is involved in regulating cold, ABA, salt, H_2_O_2_, and drought stresses (Li et al. 2023).

Additionally, xanthoxin dehydrogenase and carotenoid cleavage dioxygenase 4 (CCD4), both of which contribute to carotenoid biosynthesis, showed high expression levels. Xanthoxin dehydrogenase catalyzes the conversion of xanthoxin to abscisic aldehyde, modulating ABA levels (Endo et al. 2014), while CCD4 regulates secondary metabolite production related to aroma, flavors, and colors, helping protect plants against environmental stressors. CCD4 is highly regulated in response to stress, promoting ABA synthesis and enhancing plant antioxidant activity (Dzib-Cauich et al. 2023; Tian et al. 2024).

The high expression of photosynthesis-related genes also correlates with increased expression of genes linked to carbon metabolism. Glyceraldehyde-6-phosphate dehydrogenase, which connects the Calvin cycle and glycolysis, and glucose-6-phosphate isomerase, both key enzymes in glycolysis, were upregulated under control conditions in *A. hypochondriacus*. Transcripts associated with cell wall degradation, such as alpha-L-arabinofuranosidase and UDP-glucuronate 4-epimerase, were also identified. The former enzyme oversees the debranching of complex polysaccharides, while the latter catalyzes the epimerization of UDP-α-D-glucuronic acid to UDP-α-D-galacturonic acid, a precursor to various cell-surface polysaccharides and pectin biosynthesis (Gu and Bar-Peled 2004).

Galactinol, which acts as an osmo-protectant, is involved in plant cell wall and stress responses. It is synthesized by galactinol synthase (GolS) and myo-inositol. Both GolS and inositol oxygenase transcripts were identified. GolS is a focal point in breeding programs aimed at enhancing stress tolerance (dos Santos and Esteves Vieira 2020), while inositol oxygenase helps regulate myo-inositol levels in plants, with increased activity leading to the incorporation of myo-inositol-derived sugars into cell wall polymers (Endres and Tenhaken 2009). Furthermore, caffeoyl shikimate esterase is a key enzyme in the lignin biosynthetic pathway. The transcript for alpha-trehalose-phosphate synthase was highly expressed. Trehalose is an essential metabolite necessary for plant growth and development; it promotes stress tolerance, resilience, and increased crop yield (Kerbler et al. 2023).

Thiamine is crucial for plants, acting as an essential coenzyme in key metabolic processes, such as photosynthesis and respiration. It also supports defense mechanisms and stress resistance (Fitzpatrick and Chapman 2020). Several isoforms of phosphomethylpyrimidine synthase (PMPS), known as ThiC, have been identified as highly up-regulated in *A. hypochondriacus* under control conditions. ThiC catalyzes the conversion of 5-aminoimidazole ribonucleotide (AIR) to 4-amino-5-hydroxymethyl-2-methyl pyrimidine phosphate (HMP-P), a complex rearrangement involved in thiamine biosynthesis (Sharma et al. 2024).

In cluster 2, under control conditions, transcripts related to glucose and amino acid metabolism were highly expressed in *A. hypochondriacus*. This includes genes involved in glycolysis, such as phosphoglycerate kinase. Moreover, this cluster contains several isoforms of cystathionine gamma synthase, which is involved in the metabolism of sulfur amino acids (Bloch et al. 2021; Liu et al. 2022). It also includes a serine acetyltransferase, which catalyzes a key step in cysteine production, and sulfite oxidase, which encodes an enzyme essential for detoxifying excess sulfite (Muñoz-Vargas et al. 2025).

### Phosphonate metabolism, control of membrane integrity, and detoxification of ROS play a significant role in *A. hybridus* protection against salt stress

Water deficit and salt stress adversely affect phosphorus bioavailability, which makes it challenging for crops to absorb this essential nutrient (Dey et al. 2021). Our data indicate that *Amaranthus hybridus* under salt stress employs mechanisms to sequester phosphate through the metabolism of phosphonates and phosphinates. This metabolic pathway is downregulated under normal conditions and during water deficit. The phosphorus released from the degradation of phosphonate compounds could meet the phosphorus requirements of plants (Yu et al. 2013). Furthermore, phosphonates like methylphosphonate and 2-hydroxyethylphosphonate can be integrated into membrane lipid fractions, providing resistance against hydrolytic enzymes produced by predators (Kafarski 2020). We observed that methylphosphonate was present in *Amaranthus hypochondriacus* plants subjected to water deficit. Under salt stress, the predominant forms detected were *O*-ethyl *S*-vinyl methylphosphonothioate and butylphosphonic acid. Notably, phosphinic acid, specifically diethyl and methyl esters, were identified only in *A. hybridus* under salt stress. Transcriptome analysis revealed differential expression of pyrophosphate phosphohydrolase, a key enzyme in phosphonate metabolism. We also detected other phosphatases, such as bi-functional purple acid phosphatase, which plays a crucial role in phosphate remobilization (Bhadouria and Giri 2022), within our transcriptome data. Overall, phosphonate metabolism and related compounds may be strategies that amaranths use to protect their cell membranes and serve as a phosphorus storage source during challenging environmental conditions.

*A. hybridus* showed various mechanisms to protect its membranes and maintain cell membrane properties under salt stress conditions, notably through the upregulation of lysophospholipid acyltransferase-1 (LPLAT). LPLAT is associated with membrane asymmetry and diversity, which play a critical role in controlling the biochemical, biophysical, and structural properties of cell membranes (Pabst and Keller 2024).

Additionally, we observed high levels of expression of genes involved in steroid biosynthesis in *A. hybridus* under salt stress. Sterols are crucial for maintaining membrane integrity and serve as precursors for cellulose biosynthesis in plants (Peng et al. 2002). The primary compounds identified in steroid biosynthesis were squalene and chondrillasterol, which exhibited differential accumulation between species and under stress conditions. DEGs related to the biosynthesis of these phytosterols included 7-dehydrocholesterol reductase and cycloartenol-C-24-methyltransferase, both of which are essential for the synthesis of cholesterol and campesterol. Furthermore, monomethyl pimelate, a precursor of biotin, was detected via GC–MS. Biotin is a critical cofactor that catalyzes the first rate-limiting step of fatty acid biosynthesis and has been linked to resistance to carbonate stress (Wang et al. 2020). Another fatty acid-related compound identified was dodecyl acrylate (also known as stearyl acrylate). This fatty acid derivative was found in all samples, suggesting an important mechanism for modulating the physical properties of plasma membranes (Lomège et al. 2019).

Serine hydroxymethyltransferase 4 (SHMT) is an enzyme involved in one-carbon metabolism pathways and essential for plant growth and adaptation. Heterologous expression of cotton SHMT (GhSHMT11s) in *Arabidopsis* improved salt tolerance by reducing reactive oxygen species accumulation (Yang et al. 2024). Lipoxygenase-6 facilitates jasmonic acid (JA) production under salt stress, and JA increases were associated with improved salt tolerance by activation of antioxidant enzymes, enhanced Na^+^ extrusion, and reinforced membrane stability (Li et al. 2025). Thus, lipoxygenase-6 is crucial for plant responses to stress, particularly osmotic and drought stress, in which it plays a key role in producing signaling molecules called oxylipins, with a-linolenic acid as substrate (Li et al. 2025). Peroxidase-56 (a class III peroxidase) is a key enzyme in detoxifying harmful ROS and ROS-mediated signaling pathways that help plants acclimate to adverse conditions.

Another relevant gene found to be highly expressed encodes peroxisomal glycerate dehydrogenase HPR, the up-regulation of which has been associated with increased photorespiratory flux. Alanine aminotransferase 2 is important during hypoxia, nitrogen use efficiency, and abiotic stress tolerance by conserving carbon and nitrogen as alanine (Agrawal et al. 2024).

Riboflavin enhances salinity stress tolerance by lowering malondialdehyde and hydrogen peroxide (Jiadkong et al. 2024). Within the riboflavin biosynthetic pathway, the transcript 5-amino-6-(5-phospho-D-ribitylamino)uracil phosphatase was highly expressed.

Glutathione in *A. hybridus* plays an important role in regulating reactive oxygen species during salt stress. This tripeptide, composed of glutamate, cysteine, and glycine, acts as a reservoir for amino acids. Glutathione hydrolase is important in plant stress responses because it facilitates the recycling of amino acids. Additionally, glutathione 5'-adenylylsulfate reductase is relevant for stress responses because it plays a key role in sulfate assimilation, which is essential for cysteine production. Moreover, glutamate decarboxylase, another enzyme identified, converts glutamate into gamma-aminobutyric acid (GABA), which accumulates when phosphate (Pi) is limited, providing the plant with alternative carbon sources.

### Branched-chain amino acids and raffinose family oligosaccharides are involved in the response of *A. hypochondriacus* to water deficit and salt stress

The remarkable adaptability of *A. hypochondriacus* to abiotic conditions is likely due to the high expression levels of genes associated with the MAPK signaling pathway. This pathway is crucial for plant growth and development and plays a significant role in responding to both abiotic and biotic stresses through interactions with reactive oxygen species (ROS) and abscisic acid (ABA) levels (Singh et al. 2019). Among the DEGs, we identified genes related to phosphate signaling, encoding several isoforms of protein phosphatase 2 C (AIP1, A, ABI1). Protein phosphatases are serine/threonine-protein phosphatases involved in signal transduction pathways, including MAPK pathways, and can interact with several other kinases. They are crucial in plant responses to drought and salinity and negative regulators of ABA signaling (Fuchs et al. 2012). Transcriptome analysis also revealed the presence of a *Shewanella*-like phosphatase, a tyrosine-specific phosphatase that regulates sucrose non-fermenting-1-related protein kinases 2 (SnRK2s). SnRK2s are regulators of the plant stress response activated by drought and salinity (Krzywińska et al. 2016).

β-Alanine is a non-proteinogenic amino acid precursor of pantothenic acid, which, in turn, is a precursor of coenzyme A and participates in plant stress responses. β -Alanine is also a precursor of betaine and plays an important role in lignin biosynthesis and in the production of many secondary metabolites. In addition, the branched-chain amino acid (BCAA) degradation pathway can provide precursors for β-alanine. Alanine-glyoxylate aminotransferase 2 homolog 1, which, together with BCAA aminotransferase 2, through the propionate pathway maintains β-alanine homeostasis (Wu et al. 2025b). BCAAs function as scavengers of reactive oxygen species and BCAA aminotransferase 2, the enzyme responsible for BCAA degradation, has been shown to positively regulate salt stress tolerance by promoting the synthesis of vitamin B5 (Sun et al. 2024).

*A. hypochondriacus’* response to stress was characterized by the up-regulation of genes related to the raffinose family oligosaccharides (RFOs) biosynthesis, sucrose synthase, UDP-glucose 4-epimerase, and 4-coumarate-ligase enzyme, enzymes that work together to direct glucose towards lignin biosynthesis (Vanholme et al. 2019). In this sense, peroxidase 4-like (GPX4), a key seleno-protein antioxidant enzyme, participates in metabolic pathways involved in lignin monomer formation (Vanholme et al. 2019; Weaver and Skouta 2022). Under water deficit and salt stress, *A. hypochondriacus* responds by activating genes involved in cell wall lignification and by increasing levels of osmo-regulatory compounds through RFO accumulation.

We identified several transcripts related to heat shock proteins, including Hsp70 kDa and the 17.4 kDa class II. Hsp90 has several functions: it forms complexes with E3 ligases to promote protein degradation, stabilizes the auxin receptor TIR1, activates ABA biosynthesis, enhances insect resistance through JA signaling, and protects chloroplast DNA (Wu et al. 2025a).

Throughout their evolutionary history, wild relatives of crop plants have adapted to survive in adverse environments, continually evolving to thrive under harsh conditions. This raises the expectation that *A. hypochondriacus*, the "domesticated" and most cultivated species, would be less tolerant of stress. However, it is suggested that *A. hypochondriacus* may have originated from the crossbreeding of *A. hybridus*, a wild species adapted to salt-stress conditions, and *A. powellii*, which is adapted to water-deficit conditions (Sauer 1967; Espitia-Rangel et al. 2012; Stetter and Schmid 2017). As a result, *A. hypochondriacus* may have inherited the ability to withstand harsh conditions from its ancestors. Based on our transcriptome analyses, the tolerance of *A. hybridus* to high salinity is attributed to a constitutively active protection mechanism. In contrast, the tolerance of *A. hypochondriacus* to both salt and water deficit stress comes from a combination of this inherited protection strategy from *A. hybridus* and its ability to modify its transcriptional landscape. Constitutively active protection strategies have been documented in plants that survive rapid water loss, such as desiccation-tolerant bryophytes (Proctor et al. 2007).

## Supplementary Information

Below is the link to the electronic supplementary material.

Supplementary file1 (DOCX 2328 KB)

Supplementary file2 (XLSX 1823 KB)

Supplementary file3 (XLSX 1946 KB)

Supplementary file4 (XLSX 59 KB)

## Data Availability

The fastq files of the transcriptomic experiments generated in this work were deposited in the NCBI GEO (GSE288330) (https://www.ncbi.nlm.nih.gov/geo/query/acc.cgi?acc=GSE288330).

## Abbreviations

BCAA: Branched-chain amino acids
DEGs: Differentially expressed genes
MAPK: Mitogen-activated protein kinase
RFO: Raffinose family oligosaccharides
