# Supplementary material for: Transcriptome and metabolite profiles reveal differential molecular responses of wild and cultivated amaranth species to water deficit and salt stress
Source: Planta. 2026 Jan 29;263(3):59. doi: 10.1007/s00425-026-04927-x (PMC12855421; doi:10.1007/s00425-026-04927-x)
Supplement: Supplementary file 1 — Supplementary file1 (DOCX 2328 KB) [file 425_2026_4927_MOESM1_ESM.docx]

**Supplementary Information**

**Table S1** Summary of the transcriptome assembly statistics of *A. hybridus* and *A. hypochondriacus*

**Table S2** Identified compounds by GC-MS in amaranth leaf from control plants and plants subjected to water deficit and salt stress conditions

**Fig. S1** Number of reads per library under water deficit or salinity stress.

**Fig. S2** GO terms are enriched from genes differentially regulated both in salinity stress and water deficit.

**Fig. S3** GO Biological Process terms enriched in the clusters formed by ortholog genes expression in TPM of *A. hybridus* and *A. hypochondriacus*.

**File S1** Gene expression in *A. hybridus* under stress.

**File S2** Gene expression in *A. hypochondriacus* under stress.

**File S3** HMPorthologs and expression level.

**Supplementary Information, Tables S and Figs S**

**Transcriptomic and metabolite profiles reveal differential molecular responses of wild and cultivated amaranth species to water deficit and salt stress**

**Table S1** Summary of transcriptome assembly for *A. hybridus* and *A. hypochondriacus*

|  | *Amaranthus* | |
| --- | --- | --- |
|  | *hybridus* | *hypochondriacus* |
| Raw reads | 204,255,735 | 150,590,018 |
| Filtered reads | 204,094,843 | 150,482,640 |
| Mapped unique reads | 165,813,620 | 161,235,224 |
| Total Contigs | 38644 | 39725 |
| Total “Genes” | 33861 | 35100 |
| Non-Redundant “genes” | 30156 | 30946 |
| BUSCO_Euk (%) | 63.0 | 65.1 |
| Mapped reads (Bowtie %) | 76.29 – 83.09 | 87.87 – 89.63 |
| N50 | 1084 | 1133 |
| Contigs > 500 bp | 11087 | 11722 |

**Table S2** Identified compounds by GC-MS in amaranth leaves from control plants and plants subjected to water deficit and salt stress conditions

| **CAS NO.** | **RT** | **Name** | ***A. hypocho*** | | | ***A. hybri*** | | |
| --- | --- | --- | --- | --- | --- | --- | --- | --- |
|  |  |  | **C** | **S** | **WD** | **C** | **S** | **WD** |
| **Organic compounds** | |  |  |  |  |  |  |  |
| 13679-74-8 | 4.11 | 2-Acetyl-5-methylthiophene |  |  |  | **X** | **X** |  |
| 1014604 | 4.23 | Benzene, 1,3-bis(1,1-dimethylethyl)- | **X** | **X** | **X** |  | **X** | **X** |
| 7786610 | 4.55 | 2-Methoxy-4-vinylphenol | **X** | **X** | X | **X** | **X** |  |
| EPA-331582 | 5.33 | 2,4-Difluorobenzoic acid, 2-formyl-4,6-dichlorophenyl ester |  | **X** | X | **X** |  | **X** |
| 437-72-9 | 7.16 | Desaspidinol (organic compound) | **X** | X | X |  | **X** | **X** |
| **Flavonoids** | |  |  |  |  |  |  |  |
| 96764 | 5.37 | 2,4-Di-tert-butylphenol- Phenol | **X** | **X** | X | **X** | **X** | **X** |
| 82304-66-3 | 6.90 | 7,9-Di-tert-butyl-1, oxaspiro (4,5)deca-6,9-diene-2,8-dione | **X** | X | X | **X** | **X** | **X** |
| 150867 | 7.49 | Phytol diterpente | **X** | X | X | **X** | **X** | **X** |
| 504961 | 6.59 | Neophytadiene diterpene | **X** | **X** |  | **X** | **X** | **X** |
| 16022041 | 7.46 | Bicyclo[3.1.1]heptane, 6,6-dimethyl-3-methylene (β-**pinene) geranyl compound** |  | **X** |  | **X** |  |  |
| EPA-154772 | 7.95 | α-D-Mannofuranoside, O-geranyl  terpene glycoside, antimicrobial |  | X | X |  |  |  |
| **Alkanes, Lipids** | | |  |  |  |  |  |  |
| 112709 | 4.45 | n-Tridecan-1-ol  **(alkane)** |  |  | X |  | **X** |  |
| 2437561 | 4.84 | 1-Tridecene **alkene** | **X** | **X** | X | **X** | **X** | **X** |
| 112549 | 4.93 | Dodecanal **alkene** |  | **X** |  |  | **X** |  |
| 112538 | 5.19 | 1-Dodecanol (**lauric alchocol)** | **X** | **X** |  |  | **X** |  |
| 294622 | 5.19 | Cyclodecane **cycloalkane** |  |  | X |  |  | **X** |
| 112185 | 5.32 | 1-Dodecanamine, N,N-dimethyl- | **X** | **X** |  |  | **X** | **X** |
| 143077 | 5.55 | Dodecanoic acid (**lauric acid)** |  | **X** | X |  |  |  |
| 14620521 | 5.60 | Dodecane, 1,1-dimethoxy- |  | **X** |  |  |  |  |
| 112663 | 5.73 | Lauryl acetate |  | **X** |  |  |  |  |
| 124254 | 5.76 | Tetradecanal **(myristyl aldehyde)** |  |  |  |  | **X** |  |
| 2156970 | 6.06 | Dodecyl acrylate **(n-lauryl acrylate)** | **X** | **X** | X | **X** | **X** | **X** |
| 112754 | 6.10 | 1-Tetradecanamine, N,N-dimethyl | **X** | **X** | X | **X** | **X** |  |
| 124107 | 6.18 | Methyl tetradecanoate **(myristic acid methyl ester)** |  | X |  |  | **x** | **X** |
| 112378 | 6.30 | Undecanoic acid **(antifungal)** |  |  |  | **X** |  |  |
| 544638 | 6.31 | Tetradecanoic acid **(myristic acid)** |  | **X** | X |  | **X** | **X** |
| 20291401 | 6.34 | Monomethyl pimelate **(alkyl ether)** |  |  |  | **X** |  |  |
| 54410-98-9 | 6.38 | 1-Nonene, 4,6,8-trimethyl (**terpene)** |  |  |  |  | **X** | **X** |
| 295023 | 6.74 | Cyclotridecane **(cycloalkane)** | **X** |  | X |  |  |  |
| 112696 | 6.82 | Dimethyl palmitamine |  | X | X |  | **X** |  |
| 112390 | 6.89 | Hexadecanoic acid, methyl ester | **X** | X | X | **X** | **X** | **X** |
| 57103 | 7.00 | n-Hexadecanoic acid **(palmitic acid)** | **X** | X | X | **X** | **X** | **X** |
| 6380718 | 7.19 | Propanoic acid, 3-mercapto-, dodecyl ester | **X** |  |  | **X** | **X** |  |
| 142916 | 7.20 | Isopropyl palmitate |  | X |  |  |  |  |
| 2566974 | 7.44 | 9,12-Octadecadienoic acid, methyl ester, (E,E)- (**linolelaidic acid methyl este**r) | **X** |  | X | **X** |  | **X** |
| 301008 | 7.46 | 9,12,15-Octadecatrienoic acid, methyl ester, (Z,Z,Z)- **(methyl a-linolenate)** |  |  |  | **X** |  | **X** |
| 112618 | 7.52 | Methyl stearate | **X** | X | X | **X** | **X** | **X** |
| 57114 | 7.63 | Octadecanoic acid **(stearic acid)** | **X** | X | X | **X** | **X** | **X** |
| 1120167 | 7.70 | Dodecanamide (**lauric acid amide)** |  |  | X |  |  |  |
| 629549 | 7.70 | Hexadecanamide **(palmitic acid amide)** | **X** | X | X | **X** |  | **X** |
| 301020 | 8.23 | 9-Octadecenamide, (Z)- **(oleic acid amide)** | **X** |  |  |  | **X** | **X** |
| 646139 | 8.28 | Octadecanoic acid, 2-methylpropyl ester (**isobutyl stearate)** |  |  | X | **X** |  |  |
| 23470000 | 8.62 | Hexadecanoic acid, 2-hydroxy-1-(hydroxymethyl)ethyl ester **(DAG)** | **X** | X | X | **X** | **X** | **X** |
| 58594459 | 9.11 | 13-Octadecenal, (Z) **(alkane)** |  |  |  | **X** |  |  |
| 112845 | 9.36 | 13-Docosenamide, (Z)- **(antimicrobial)** | **X** | X | X | **X** | **X** | **X** |
| **Hydrocarbons and sterols** | | |  |  |  |  |  |  |
| 111024 | 9.50 | Squalene | **X** | X | X | **X** | **X** | **X** |
| 7616220 | 10.21 | α-Tocopherol | **X** | X | X | **X** | **X** | **X** |
| 59029 | 10.50 | Vitamin E | **X** | X | X | **X** | **X** | **X** |
| 481174 | 11.34 | Chondrillasterol | **X** | X | X | **X** | **X** | **X** |
| 35933007 | 11.64 | Stigmast-7-en-3-ol, (3á,5à)- | **X** | X | X | **X** | **X** | **X** |
| **Phosphonates** | |  |  |  |  |  |  |  |
| EPA-298385 | 5.08 | 3,3,5,5-Tetramethylcyclohexyl methylphosphonofluoridate |  | **X** |  |  |  |  |
| EPA-322851 | 6.40 | Butylphosphonic acid, butyl isohexyl ester | **X** | **X** |  |  |  |  |
| 59651637 | 6.74 | Methylphosphonic acid, didecyl ester |  |  | **X** |  |  |  |
| 128869818 | 9.14 | O-Ethyl S-vinyl methylphosphonothioate |  | X |  | **x** |  |  |
| EPA-306035 | 9.13 | Phosphinic acid, diethyl-, methyl ester |  |  |  |  | **X** | **X** |

RT=retention time; C=control conditions; S=salinity; WD=water deficit

**Fig. S1** Number of reads per amaranth species. Ahybr=*Amaranthus hybridus*; Ahypo=*Amaranthus hypochondriacus.* Libraries of control plants (C); under water deficit (WD), and salinity stress (S)

**Fig. S2** GO terms enriched from genes regulated both in salinity and water deficit. **A** GO terms enriched from genes regulated by salinity stress and water deficit in *A. hybridus*. **B** GO terms enriched from genes up regulated by salinity stress and water deficit in *A. hypochondriacus*. **C** GO terms enriched from genes down regulated by salinity and drought stress in *A. hypochondriacus*. S_WD, genes regulated both in salinity stress and water deficit. S, genes regulated in salinity. WD, genes regulated in water deficit


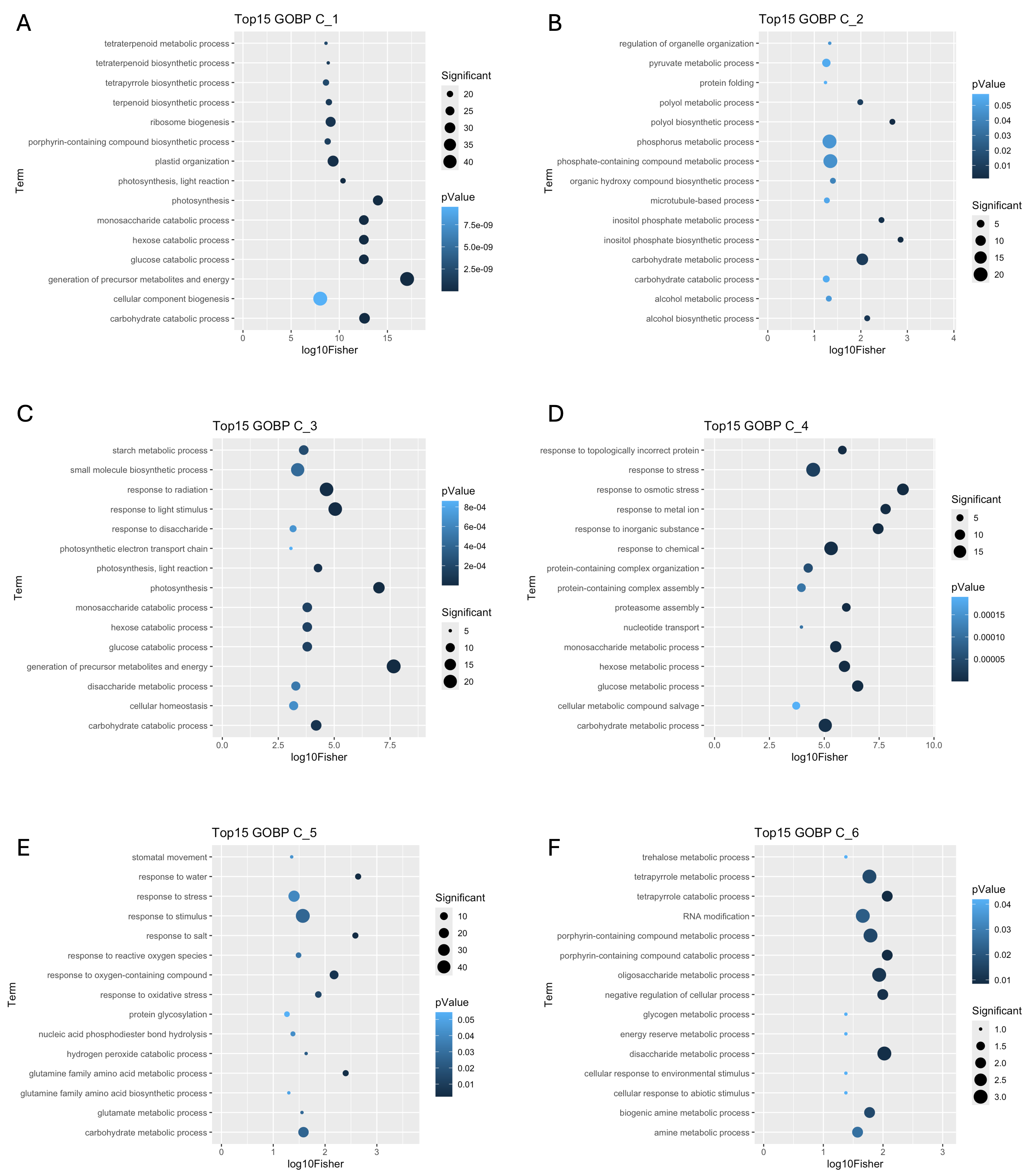


**Fig. S3** GO Biological Process terms enriched in the clusters formed by orthologs genes expression in TPM of *A. hybridus* and *A. hypochondriacus*. The graphs show the 15 most significantly enriched terms for each cluster (1-6) in comparison of expression profiles of *A. hybridus* and *A. hypochondriacus* orthologs. **A-F** Clusters 1-6, as indicated. The size of the black circles represents the number of genes in th GO term, *P* values bigger to lowest are colored as blue to dark. Axis X values are the log10 (*P* values).
